# Management of potentially curable colorectal lung metastases with synchronous systemic therapy and percutaneous image-guided thermal ablation

**DOI:** 10.1080/07853890.2025.2612393

**Published:** 2026-01-05

**Authors:** Hongjie Fan, Bufu Tang, Xiangjun Dong, Yulan Zeng, Xinyue Gu, Xiangwen Xia, Jihong Hu, Cheng Wan, Rong Ding, Xinghai Li, Fenhua Zhao, Chunlong Fu, Jiangping Cun, An Li, Xuancheng Xie, Shufeng Xu, Kun Qian, Xuefeng Kan, Chuansheng Zheng

**Affiliations:** ^a^ Department of Radiology, Union Hospital, Tongji Medical College, Huazhong University of Science and Technology, Wuhan, China; ^b^ Hubei Provincial Clinical Research Center for Precision Radiology & Interventional Medicine, Wuhan, China; ^c^ Hubei Provincial Key Laboratory of Molecular Imaging, Wuhan, China; ^d^ Department of Radiation Oncology, Zhongshan Hospital, Fudan University, Shanghai, China; ^e^ Cancer Center, Union Hospital, Tongji Medical College, Huazhong University of Science and Technology, Wuhan, China; ^f^ Department of Minimally Invasive Intervention, The First Affiliated Hospital of Kunming Medical University, Kunming, China; ^g^ Department of Minimally Invasive Intervention, Yunnan Cancer Hospital, The Third Affiliated Hospital of Kunming Medical University, Kunming, China; ^h^ Department of Minimally Invasive Intervention, Ganzhou People’s Hospital, Ganzhou, China; ^i^ Department of Radiology, Affiliated Dongyang Hospital of Wenzhou Medical University, Dongyang, China; ^j^ Department of Radiology, Second Affiliated Hospital of Kunming Medical University, Kunming, China; ^k^ Department of Interventional, Xiangyang Central Hospital, Affiliated Hospital of Hubei University of Arts and Sciences, Xiangyang, China; ^l^ Department of Radiology, The First People’s Hospital of Yunnan Province, Kunming, China; ^m^ Department of Radiology, Quzhou Hospital Affiliated to Wenzhou Medical University, Quzhou, China

**Keywords:** Colorectal lung metastases, image-guided thermal ablation, systemic therapy, survival outcomes, local tumor progression

## Abstract

**Objective:**

To assess the survival benefit of synchronous systemic therapy plus thermal ablation (TA) in oligometastatic colorectal lung metastases (CRLM) and identify independent prognostic factors.

**Background:**

Optimizing the integration of systemic therapy and TA for potentially curable CRLM remains a significant clinical challenge.

**Methods:**

This study employed a retrospective cohort design, including 326 patients who underwent TA treatment at six tertiary medical centers from March 2014 to October 2022. Patients were categorized into synchronous therapy, upfront ablation, delayed ablation, and no systemic therapy groups based on the timing of systemic therapy relative to TA. Kaplan–Meier analysis and log-rank tests were used to assess survival outcomes.

**Results:**

Synchronous systemic therapy yielded the longest median progression-free survival (PFS) (22.0 months) and overall survival (OS) (61.3 months) compared to delayed ablation (13.0 and 49.2 months, respectively) and no systemic therapy (11.9 and 29.3 months, respectively) (all *p* < 0.05). Synchronous systemic therapy was an independent protective factor for PFS [hazard ratio (HR) = 0.493] and OS (HR = 0.211). Independent risk factors for local tumor progression included tumor size ≥3 cm (HR = 1.75) and peridiaphragmatic location (HR = 1.48). For PFS, independent predictors included tumor numbers (*p* < 0.001), synchronous metastases (HR = 1.431), and extrapulmonary metastases (*p* = 0.001). OS was adversely influenced by tumor burden (*p* < 0.05), extrapulmonary metastases (*p* < 0.001), and mediastinal lymph node involvement (HR = 1.518).

**Conclusions:**

Synchronous systemic therapy combined with TA significantly enhances PFS and OS in potentially curable oligometastatic CRLM patients.

## 1. Background

Colorectal cancer (CRC), the second leading cause of cancer-related mortality for men and women combined globally, presents a critical clinical challenge due to its propensity for pulmonary metastases—second only to hepatic dissemination in frequency [1,2]. In patients with potentially curable colorectal cancer lung metastases (CRLM), the primary therapeutic goal is to achieve a state of no evidence of disease (NED) through definitive local treatment [3]. Accordingly, the optimal management of CRLM requires a multidisciplinary approach to tailor individualized treatment strategies and improve clinical outcomes.

This necessitates a comprehensive assessment of critical factors—including metastatic burden (number and size of lesions), anatomical distribution, and the presence of extrapulmonary disease—to inform multidisciplinary decision-making. Although pulmonary metastasectomy has shown well-established oncologic benefits in carefully selected patient cohorts [4,5], image-guided thermal ablation (IGTA) has emerged as a minimally invasive alternative for patients with oligometastatic disease, typically defined as involvement of ≤2 metastatic organs or ≤5 pulmonary lesions [3,6–9].

Advances in systemic therapies—including immunotherapy, targeted agents, and combination chemotherapy—have significantly transformed the therapeutic landscape of CRC [10]. Current guidelines from the European Society of Medical Oncology (ESMO) recommend integrating local ablative therapies with systemic treatments, particularly for patients with oligometastatic disease [11]. However, the optimal sequencing of systemic therapy in relation to IGTA remains a critical yet unresolved clinical question [12,13]. Although peri-ablative systemic therapy holds promise for improving long-term outcomes by targeting micrometastatic disease and providing insights into tumor biology, definitive evidence supporting its routine implementation is currently insufficient.

This study utilizes multicenter clinical data to assess the oncologic efficacy of IGTA in combination with systemic therapies for CRLM. By stratifying patients according to the timing of systemic therapy—categorized as absent, administered prior to IGTA, during the peri-ablative period, or following IGTA—the study seeks to elucidate the impact of therapeutic sequencing on tumor control and survival outcomes. The results are anticipated to inform clinical decision-making and support evidence-based strategies aimed at improving prognosis in this complex patient population.

## 2. Methods

### 2.1. Patients

This study was conducted in accordance with the principles of the Declaration of Helsinki and was approved by the Institutional Review Board of Union Hospital, Tongji Medical College, Huazhong University of Science and Technology (IRB approval number: IEC-2024-1103). Written informed consent was obtained from all participants prior to enrollment.

A comprehensive review of clinical and imaging data was conducted for patients with CRLM who underwent percutaneous IGTA between March 2014 and October 2022 at six academic centers. Inclusion and exclusion criteria were rigorously defined to ensure analytical reliability and relevance (Table 1). It is noteworthy that systemic therapy records around the IGTA procedure were unavailable in 92 patients (23.8%). These patients were retained in the overall survival analyses but were excluded from subgroup analyses requiring systemic therapy information.

**Table 1.** The inclusion and exclusion criteria for this study.

Inclusion criteria	Exclusion criteria
(1) Histologically confirmed CRC.	(1) Age <18 or >85 years.
(2) Underwent CT-guided RFA or MWA.	(2) Child-Pugh C liver function status.
(3) Maximum CRLM diameter < 50 mm and ≤ 5 lesions per patient.	(3) Insufficient image quality to assess lesions.
(4) Complete clinical and imaging data available.	(4) History of other malignant tumors within the preceding 5 years.
(5) R0 resection of the primary tumor.	(5) Uncontrolled extrapulmonary metastases.
(6) No prior IGTA performed for CRLM.	(6) Pregnancy or breastfeeding women.
(7) An ECOG performance status 0–2.	(7) Follow-up duration of <36 months.
(8) Oligometastatic CRC.	(8) Severe pulmonary dysfunction.

*Note:* CRC, colorectal cancer; CRLM, colorectal cancer lung metastasis; CT, computed tomography; RFA, radiofrequency ablation; MWA, microwave ablation; IGTA, image-guided thermal ablation; ECOG, Eastern Cooperative Oncology Group.

### 2.2. Study design

Patient characteristics and clinical variables, including age, sex, primary tumor location, treatment history (e.g. pneumonectomy, chemotherapy, radiotherapy, immunotherapy, targeted therapy), body mass index (BMI), comorbidities, plasma levels of carcinoembryonic antigen (CEA), carbohydrate antigen 125 (CA125), carbohydrate antigen 19-9 (CA19-9), tumor differentiation, Eastern Cooperative Oncology Group (ECOG) performance status, TNM staging, disease-free interval (DFI), synchronous metastases, tumor size and number, extrapulmonary metastasis, and IGTA parameters (e.g. ablation type, duration, power, temperature, puncture depth), were extracted from historical records and electronic databases by trained clinicians.

Imaging data were reviewed by a panel of ten radiologists with over 10 years of expertise in medical imaging, independent of the IGTA procedures. Computed tomography (CT) measurements were based on 2-mm slice thickness reconstructions from the Picture Archiving and Communication System (PACS). Evaluated features included lesion count, maximum three-dimensional diameter, anatomical location (e.g. pulmonary distribution, proximity to vessels, bronchi, pleura, and diaphragm), and CT findings such as attenuation values, ground-glass opacities, solid lesions, calcifications, emphysema, and inflammatory changes.

Following National Comprehensive Cancer Network (NCCN) guidelines and prior studies, lesions in the medial two-thirds of the lung were categorized as central, while those in the outer one-third were classified as peripheral [14,15]. Tumors in contact with pulmonary vessels or bronchi ≥3 mm in diameter were considered perivascular or peribronchial, respectively [16]. Lesions within 1 cm of the pleura (mediastinal, parietal, interlobar) or diaphragm were deemed adjacent. Lymph nodes were positive if their short-axis exceeded 1 cm, had >1 mm extranodal extension, or malignancy was pathologically confirmed [17].

The interval between CRC resection and pulmonary metastasis detection was recorded. All patients with oligometastatic CRC had effective control of their extra-pulmonary metastatic lesions, and pulmonary metastases were considered potentially curable based on discussion by a multidisciplinary team of experts. Therefore, the goal of IGTA for CRLM was to achieve NED. Treatment plans were individualized following evaluation by a multidisciplinary tumor board comprising thoracic surgeons, interventional radiologists, oncologists, radiologists, and radiation oncologists. Strategies included IGTA and systemic therapy, considering patient preferences. Based on the timing of IGTA in relation to systemic therapy, the patients were categorized into the following four cohorts: (1) No systemic therapy within 3 months before or after IGTA; (2) Synchronous therapy (systemic therapy administered both before and after IGTA, with more than two cycles in total); (3) Pre-IGTA ablation (systemic therapy initiated after IGTA, with more than two cycles of adjuvant treatment); or (4) Delayed IGTA (IGTA performed after at least two cycles of systemic therapy) [12]. Systemic regimens included FOLFOX, FOLFIRI, CAPOX, and FOLFOXIRI, with or without bevacizumab or cetuximab.

### 2.3. IGTA procedure

CRLM diagnosis was confirmed *via* biopsy, pulmonary metastasis resection, or new/enlarged nodules identified on dynamic contrast-enhanced CT (DCE-CT). Preoperative evaluations included DCE-CT, blood tests, liver and renal function assessments, coagulation profiles, ECG, and pulmonary function tests. Interventional radiologists explained procedural risks, potential complications, and benefits before obtaining written informed consent.

Appropriate ablation needles (monopolar or multi-electrode radiofrequency: 14 Ga or 16 Ga; microwave: 17 Ga) were selected based on tumor size and location. Patients were positioned (supine, lateral, or prone) according to tumor distribution. A team of 2–3 interventional radiologists, each with at least 10 years of oncology experience, performed the IGTA procedures. Patients remained conscious under sedation and analgesia, with continuous monitoring of vital signs. CT imaging guided needle placement, target identification, and ablation. Post-procedure CT scans (immediate and 24 h later) assessed tumor coverage and complications, classified as minor or major per the Society of Interventional Radiology Clinical Practice Guidelines [18].

### 2.4. Follow-up

At 3–4 weeks post-IGTA, DCE-CT was performed to assess tumor coverage, with complete ablation considered technically successful. Repeat IGTA was performed for treatment failures, with technical success rates documented. Local tumor progression (LTP) was defined as recurrence within 1 cm of the ablation zone >4 weeks post-procedure. For LTP, repeat IGTA or multimodal treatments (e.g. radioactive particle implantation, radiotherapy, chemotherapy, targeted therapy) were utilized. Disease control was monitored with DCE-CT every 3 months during the first year and every 6 months for the subsequent two years. Follow-up frequency and modality depended on treatment response, patient compliance, and baseline characteristics, with telephone follow-ups supplementing in-person visits. The follow-up period extended from March 2014 to December 2024 and was conducted solely for retrospective outcome assessment, with no prospective interventions performed. Given the multicentre and long-term nature of the study, all cohorts were encompassed within the defined data collection and follow-up timeframe. Primary endpoints included LTP-free survival (LTPFS), PFS, and OS. Secondary endpoints encompassed prognostic factors, technical success rates, and complications.

### 2.5. Statistical analysis

Statistical analyses were performed using Stata 17.0 (StataCorp, USA). Continuous variables with normal distributions were reported as mean ± standard deviation, while categorical variables were presented as n (%). Skewed variables were summarized as median and interquartile range (IQR). Survival outcomes (LTPFS, PFS, OS) were compared across groups using Kaplan–Meier analysis and log-rank tests. Failure estimate curves assessed cumulative LTP probabilities, while Nelson–Aalen cumulative hazard curves evaluated mortality risk accumulation over time. Smoothed hazard estimates quantified instantaneous risk changes for disease progression following IGTA. Univariate Cox regression identified potential predictors of LTPFS, PFS, and OS, which were further validated using multivariate Cox regression to adjust for confounders. A two-sided p-value <0.05 was considered statistically significant.

## 3. Results

### 3.1. Baseline characteristics

A total of 326 consecutive patients (62.3% male; mean age, 61.3 ± 9.7 years) underwent IGTA for CRLM. The rectum was the most common primary tumor site (45.4%), with moderate differentiation predominating (42.9%). Synchronous metastases were observed in 25.2% of patients, and most tumors were classified as T3 stage (54.9%). The liver was the most frequent extrapulmonary metastatic site (20.6%). Prior pulmonary metastasectomy had been performed in 8.9% of patients, with a median interval of 20 months (IQR, 25.5) between surgery and IGTA.

The median interval from primary tumor resection to lung metastases was 25 months (IQR, 21.0), with 45.4% of patients having a DFI <24 months. Across 644 metastases, the mean maximum tumor diameter was 18.1 ± 10.8 mm (range, 2.8–46.5 mm), with 39.6% of patients presenting with a single lesion. Most metastases (88.7%) were located in peripheral lung zones. Detailed demographics, clinical, and radiological characteristics are provided in Table 2.

**Table 2.** Baseline characteristics of patients undergoing IGTA for CRC lung metastases.

Variables	N (%)	Variables	N (%)
Age (years)	61.3 ± 9.7	T-stage	
Sex (male)	203 (62.3)	- T1-2	44 (13.45)
BMI (kg/m^2^)	20.9 ± 3.6	- T3	179 (54.9)
Primary tumor location		- T4	103 (31.6)
- Rectum	148 (45.4)	Tumor size (mm)	18.1 ± 10.8
- Sigmoid colon	64 (19.6)	Tumor size > 3 cm	66 (20.2)
- Descending colon	43 (13.2)	Tumor number	
- Transverse colon	21 (6.4)	- 1 tumor	129 (39.6)
- Ascending colon	50 (15.3)	- 2 tumors	102 (31.3)
Tumor differentiation		- 3 tumors	69 (21.2)
- Poor	23 (7.1)	- 4 tumors	26 (8.0)
- Poor-to-moderate	59 (18.1)	Lung location	
- Moderate	140 (42.9)	- Left lung	129 (39.6)
- Moderate-to-well	63 (19.3)	- Right lung	160 (49.1)
- Well	41 (12.6)	- Bilateral involvement	37 (11.3)
Extrapulmonary metastasis		Peridiaphragmatic tumors	29 (8.9)
- None	188 (57.7)	Near parietal pleural	145 (44.5)
- Liver	67 (20.6)	Near mediastinal pleural	51 (15.6)
- Bone	13 (4.0)	Near interlobar fissure	102 (31.3)
- Abdominal cavity	20 (6.1)	Peribronchial tumors	52 (16.0)
- Pelvic cavity	18 (5.5)	Perivascular tumors	65 (19.9)
- Other conditions	20 (6.1)	Emphysema	50 (15.3)
CEA > 20 ng/mL	42 (12.9)	Hilar lymph nodes (+)	45 (13.8)
CA19-9 > 37 U/mL	42 (12.9)	Mediastinal lymph nodes (+)	52 (16.0)

*Note:* CRC, colorectal cancer; BMI, Body Mass Index; IGTA, image-guided thermal ablation; CEA, carcinoembryonic antigen; CA19-9, carbohydrate antigen 19-9.

### 3.2. IGTA and systemic therapy

The median interval from primary tumor resection to IGTA was 28 months (IQR, 22.0). Among 305 patients treated with a single IGTA session, 37 presented with bilateral metastatic disease. Microwave ablation (MWA) was employed in 57.1% of cases, while radiofrequency ablation (RFA) accounted for 42.9%. The initial technical success rate was 97.9% (319/326), and secondary technical success reached 100% (326/326). Fractional ablation sessions, targeting 36 metastases in 21 patients, were completed within one month. The mean ablation duration was 12.2 ± 9.2 min. Median power outputs were 180 W (IQR, 110 W) for RFA and 40 W (IQR, 10 W) for MWA, with a mean puncture depth of 7.2 ± 2.0 cm. Based on the timing of systemic therapy, 103 patients underwent delayed ablation, 67 synchronous ablation, and 64 upfront ablation. Systemic therapy records were unavailable for 92 patients during the perioperative period of IGTA.

### 3.3. LTP and LTPFS

**Figure 1.**
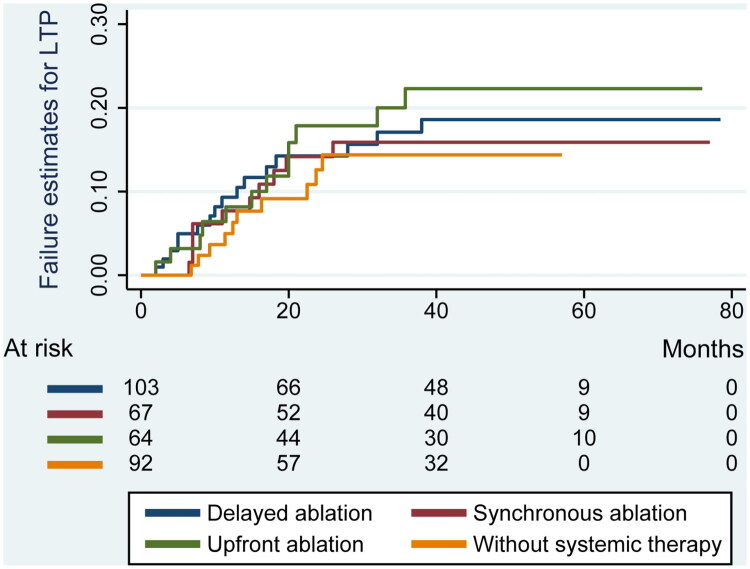
Kaplan–Meier failure estimates for LTP across groups. Pairwise comparisons using the Log-Rank test showed no statistically significant differences in LTP between the delayed ablation group, without systemic therapy group, synchronous ablation group, and upfront ablation group (all *p* > 0.20). LTP refers to local tumor progression.

During a median follow-up of 40.3 months (IQR, 31.5), LTP was observed in 48 patients. Cumulative LTP rates were 7.1% at 1 year and 14.4% at 3 years. By treatment group, LTP rates were 15.5% in the delayed ablation group, 14.9% in the synchronous ablation group, 18.9% in the upfront ablation group, and 10.9% in the no systemic therapy group (Table 3). Kaplan-Meier analysis and Cox regression revealed no significant differences in LTP or LTPFS across groups (Figure 1).

**Table 3.** Rates (standard error) of LTPFS, PFS and OS according to the type of treatment mode.

	IGTA alone (%)	Delayed ablation (%)	Synchronous ablation (%)	Upfront ablation (%)
LTPFS				
1 year	95.0 (2.4)	90.7 (3.0)	92.3 (3.3)	91.8 (3.5)
2 years	87.4 (4.0)	85.7 (3.7)	85.8 (4.4)	82.1 (5.2)
3 years	85.6 (4.3)	82.9 (4.3)	84.1 (4.6)	77.7 (5.8)
PFS				
1 year	50.0 (5.2)	52.4 (4.9)	65.7 (5.8)	62.5 (6.1)
2 years	24.8 (4.5)	28.2 (4.4)	46.3 (6.1)	37.5 (6.1)
3 years	10.9 (3.3)	20.0 (4.0)	35.8 (5.9)	26.3 (5.5)
4 years	6.2 (2.9)	18.8 (3.9)	23.7 (6.2)	24.4 (5.5)
5 years	0.0 (0.0)	13.7 (4.2)	10.2 (5.9)	24.4 (5.5)
OS				
1 year	82.6 (4.0)	84.5 (3.6)	97.0 (2.1)	87.5 (4.1)
2 years	57.6 (5.2)	66.0 (4.7)	85.1 (4.4)	70.3 (5.7)
3 years	45.7 (5.2)	58.3 (4.9)	76.1 (5.2)	60.9 (6.1)
4 years	35.1 (5.5)	51.9 (5.0)	68.6 (5.9)	57.6 (6.2)
5 years	23.4 (7.7)	41.1 (6.2)	50.8 (8.5)	48.1 (6.8)

*Note:* LTPFS, local tumor progression-free survival; OS, overall survival; PFS, progression-free survival. The data are presented as percentage (standard error).

Risk factors for LTP included tumor number, tumor size (>3 cm), and peridiaphragmatic location (all *p* < 0.001), while age, sex, synchronous metastasis, tumor differentiation, T stage, and other factors showed no significant associations with local efficacy (*p* > 0.05) (Table S1).

### 3.4. PFS

**Figure 2.**
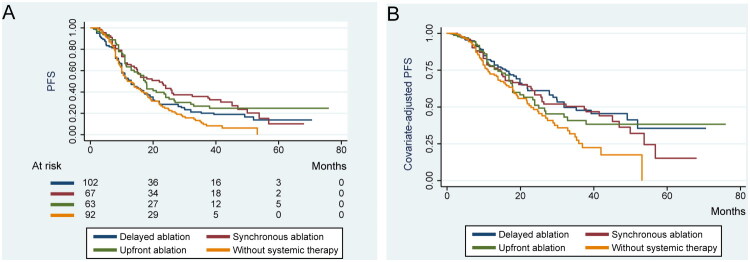
Kaplan–Meier analysis for PFS across groups. (A) Compared to patients without systemic therapy, the combination of systemic therapy with synchronous ablation (*p* < 0.001) or upfront ablation (*p* = 0.009) was associated with significantly improved PFS. (B) After adjusting for covariates using univariable and multivariable Cox proportional hazards regression, systemic therapy combined with synchronous ablation or upfront ablation remained significantly associated with better disease control (all *p* < 0.01). PFS refers to progression-free survival.

Disease progression occurred in 269 patients, with 52.8% progressing within 12 months. The median PFS was 14.8 months (95% CI, 12.595–17.065). Cumulative PFS rates at 1, 3, and 5 years were 56.4% (SE, 2.7%), 22.0% (SE, 2.3%), and 13.2% (SE, 2.4%), respectively (Table 3). Median PFS was significantly shorter in patients without systemic therapy (11.9 months, 95% CI, 8.532–15.168) compared to the synchronous ablation group (22.0 months, 95% CI, 14.309–29.691; *p* < 0.001) and the upfront ablation group (17.0 months, 95% CI, 14.387–19.613; *p* = 0.009) (Figure 2). Delayed ablation resulted in a median PFS of 13.0 months (95% CI, 10.494–15.506), which trended shorter than synchronous ablation (*p* = 0.060).

Synchronous (HR, 0.493; 95% CI, 0.341–0.712; *p* < 0.001) and upfront ablation (HR, 0.563; 95% CI, 0.391–0.810; *p* = 0.002) were protective factors for PFS, while higher tumor numbers (*p* < 0.001), synchronous metastases (*p* = 0.017), and extrapulmonary metastases (*p* < 0.001) were associated with worse PFS (Table S2).

### 3.5. OS

**Figure 3.**
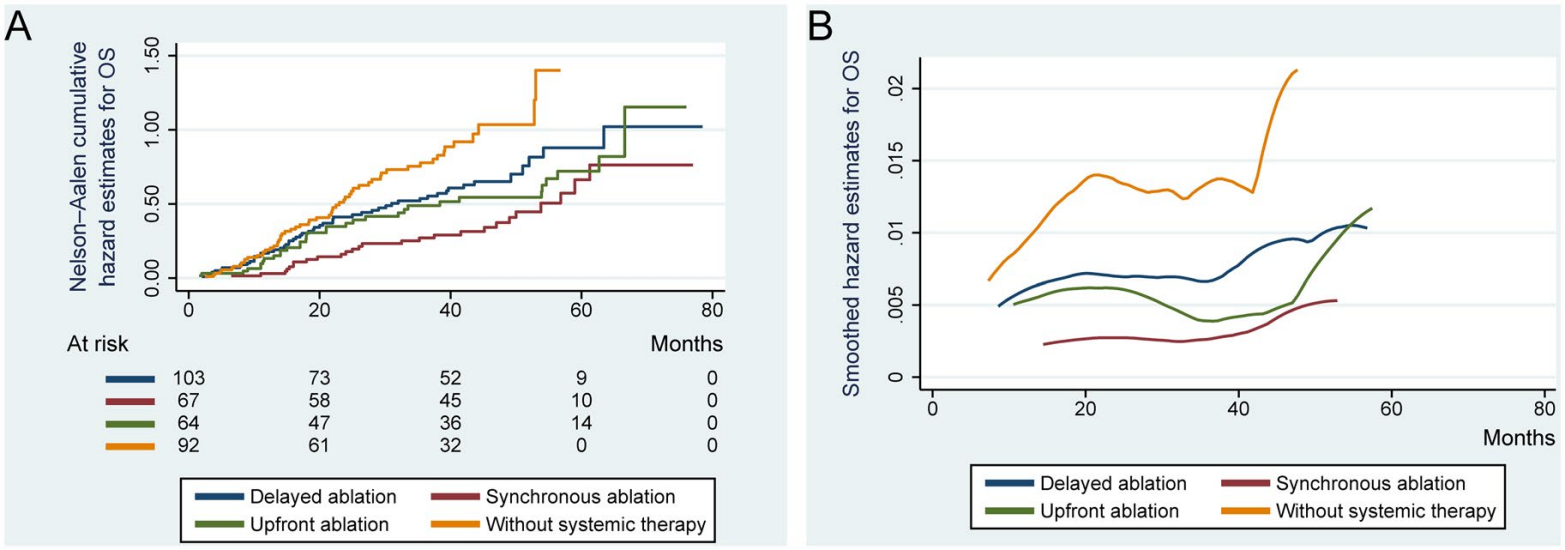
Nelson–Aalen cumulative hazard and smoothed hazard estimates for OS across groups. (A) The cumulative hazard of death in patients from the cohort with absent systemic therapy is higher than that of the other groups, particularly after 2 years of follow-up. (B) After adjusting for covariates using univariable and multivariable Cox proportional hazards regression, the dynamic changes in the mortality risk across different groups are shown: the combination of systemic therapy with synchronous ablation strategy more effectively and steadily delays the accumulation of mortality risk. OS refers to overall survival.

A total of 172 patients (52.8%) died during follow-up. Median OS was 51.0 months (95% CI, 43.505–58.495), with cumulative OS rates of 87.1% (SE, 1.8%) at 1 year, 58.9% (SE, 2.7%) at 3 years, and 40.1% (SE, 3.5%) at 5 years (Table 3). Patients without systemic therapy had a significantly shorter median OS (29.3 months) than those in the delayed (49.2 months, *p* = 0.042), synchronous (61.3 months, *p* < 0.001), and upfront (56.4 months, *p* = 0.008) ablation groups (Figure 3). Synchronous ablation was associated with longer OS than delayed ablation (*p* = 0.019). This finding was supported by univariable and multivariable Cox regression analyses (Table S3).

Independent risk factors for diminished OS included tumor burden (number and size >3 cm, *p* = 0.002 and *p* = 0.034, respectively) and mediastinal lymph node involvement (HR, 1.518; *p* = 0.033). Extrapulmonary metastases significantly worsened OS, particularly for metastases in bone (HR, 3.906), abdominal cavity (HR, 5.837), pelvic cavity (HR, 3.231), or other locations (HR, 7.516) (Table S3).

### 3.6. Subgroup analysis

Subgroup and pairwise comparisons of Kaplan–Meier curves were performed for OS and PFS according to systemic regimens and the timing of IGTA (Table 4). In the FOLFOX subgroup, patients receiving synchronous ablation showed a significantly longer estimated OS compared with those in the delayed ablation group (χ^2^ = 4.635, *p* = 0.031). In the delayed ablation subgroup, FOLFIRI achieved a significantly longer median OS than CAPOX (χ^2^ = 3.936, *p* = 0.047). Within the synchronous ablation subgroup, no significant differences in OS were observed among different chemotherapy regimens (*p* > 0.05 for all comparisons). In the upfront ablation subgroup, FOLFOXIRI resulted in a longer median OS than CAPOX (χ^2^ = 5.325, *p* = 0.021). A subgroup analysis of targeted therapy efficacy further demonstrated that, within the synchronous ablation group, patients receiving bevacizumab exhibited shorter OS and PFS than those treated with cetuximab (χ^2^ = 5.902, *p* = 0.015; χ^2^ = 8.449, *p* = 0.004, respectively). No statistically significant differences in PFS or OS were observed in pairwise comparisons among the other subgroups (*p* > 0.05 for all comparisons).

**Table 4.** Estimated PFS and OS according to systemic regimens and timing of ablation.

Ablation timing	Regimen	PFS (months, 95% CI)	OS (months, 95% CI)
Delayed ablation	FOLFOX	11.0 (7.019–14.981)	32.0 (11.062–52.938)
	FOLFIRI	12.0 (8.674–15.326)	NR
	CAPOX	10.0 (7.701–12.299)	26.4 (13.343–52.553)
	FOLFOXIRI	22.0 (14.536–29.464)	NR
	Bevacizumab	13.0 (8.932–17.068)	49.2 (38.347–60.053)
	Cetuximab	11.0 (7.728–14.272)	NR
Synchronous ablation	FOLFOX	19.67 (5.451–33.889)	NR
	FOLFIRI	16.0 (5.669–26.331)	NR
	CAPOX	16.0 (6.796–31.204)	NR
	FOLFOXIRI	22.0 (7.333–36.667)	NR
	Bevacizumab	18.0 (9.751–26.249)	53.8 (44.563–63.037)
	Cetuximab	26.0 (2.345–52.406)	NR
Upfront ablation	FOLFOX	11.5 (8.236–14.764)	54.0 (33.175–73.825)
	FOLFIRI	18.0 (7.533–28.467)	NR
	CAPOX	15.0 (8.238–21.762)	25.5 (6.933–43.967)
	FOLFOXIRI	24.0 (13.550–34.450)	NR
	Bevacizumab	20.3 (14.253–26.288)	46.3 (39.077–53.437)
	Cetuximab	32.0 (12.029–51.971)	54.6 (43.741–65.396)

*Note:* Data are presented as estimated median survival (months, 95% confidence interval). OS, overall survival; PFS, progression-free survival; NR, not reached.

### 3.7. Adverse events (AEs)

The rates of minor and major complications were 71.5% and 8.0%, respectively (Table 5). Pneumothorax, the most common AE, occurred in 160 patients, with 9 requiring aspiration and 11 requiring thoracic drainage. Pleural effusion was reported in 49 patients, with drainage required in 3 cases. Minor hemoptysis occurred in 19.6% of patients, resolving spontaneously in most cases. Two patients underwent bronchial artery embolization for hemoptysis. Other AEs included minor skin burns (1 patient) and one case of respiratory failure requiring ICU admission, with subsequent full recovery. No treatment-related deaths or permanent sequelae were reported.

**Table 5.** Adverse events (AEs) associated with image-guided thermal ablation (IGTA) for colorectal cancer lung metastases.

Event	Grade*	N (%)	Management
Pneumothorax	Overall	160 (49.1)	
	A-B	140 (42.9)	Conservative management; close monitoring
	C	12 (3.7)	needle aspiration or chest tube placement for drainage
	D	8 (2.5)	Chest tube insertion with continued monitoring for re-expansion
Pleural effusion	Overall	49 (15.0)	
	A-B	44 (13.5)	Observation
	C	4 (1.2)	Observation and symptomatic treatment; chest tube placement
	D	1 (0.3)	Chest tube placement
Subcutaneous hematoma	C	1 (0.3)	Compression dressing
Hemothorax	A	3 (0.9)	Conservative management with observation and periodic imaging
Hemoptysis	C	3 (0.9)	Pharmacological agents; BAE if bleeding persists
Nausea or vomiting	B	6 (1.8)	Preventing dehydration and electrolyte imbalance
Fever	Overall	42 (12.9)	
	A-B	40 (12.3)	Antipyretic therapy
	C	2 (0.6)	Antipyretic therapy; investigation (e.g. blood cultures, chest CT)
Pneumonia	Overall	3 (0.9)	
	C	2 (0.6)	Empiric antibiotic therapy, tailored after microbiological identification
	D	1 (0.3)	Antibiotic therapy guided by culture and sensitivity results
Respiratory failure	D	1 (0.3)	ICU admission
Skin burn	C	1 (0.3)	Medical-grade burn dressings

*Note:* AEs related to IGTA were recorded according to the Society of Interventional Radiology Clinical Practice Guidelines Classification System. AEs, adverse events; IGTA, image-guided thermal ablation; CRC, colorectal cancer; BAE, bronchial artery embolization; ICU, Intensive care unit.

## 4. Discussion

This study provides a novel perspective on the integration of systemic therapy and IGTA for CRLM, offering critical insights into the impact of treatment timing on clinical outcomes. By categorizing patients based on the temporal sequencing of systemic therapy and IGTA, this investigation uniquely elucidates how synchronization or delay influences PFS, OS, and LTPFS. Beyond survival metrics, the study also identifies independent prognostic factors for each endpoint, shedding light on tumor-specific and procedural variables that may inform future treatment strategies. These findings underscore the potential to refine therapeutic protocols for CRLM, bridging a crucial gap in ­personalized oncology care.

Our findings highlight the superior efficacy of synchronous IGTA combined with systemic therapy, which achieved the longest median PFS of 22.0 months and OS of 61.3 months compared to other treatment groups. This survival benefit suggests that concurrent systemic therapy not only addresses micro-metastases but also amplifies the immune response to IGTA-induced tumor antigen release. A preclinical study has demonstrated that IGTA can trigger immunogenic cell death, leading to the release of tumor-associated antigens and activation of dendritic cells [19]. Concurrent systemic therapy—through cytotoxic, targeted, or antiangiogenic mechanisms—may further enhance this effect by reducing immunosuppressive cell subsets and promoting T-cell-mediated cytotoxicity. This dual mechanism could underlie the synergistic benefit observed in our cohort. In this study, systemic regimens primarily comprised fluoropyrimidines combined with irinotecan or oxaliplatin, the cornerstone of chemotherapy for metastatic CRC, designed to enhance tumor response rates and prolong PFS and OS [11]. Bevacizumab enhances therapeutic outcomes by reducing tumor angiogenesis and improving perfusion, while cetuximab enhances chemosensitivity and synergizes with ablation-induced immunogenic effects in RAS wild-type CRC [20,21]. Emerging systemic therapies, including immune checkpoint inhibitors such as anti-PD-1/PD-L1 agents, are also being explored in CRC pulmonary metastases, showing promise in enhancing immune-mediated tumor control [22,23]. The integration of chemotherapy, targeted therapies, and selective use of immunotherapy strengthens systemic control while complementing IGTA’s local effects. However, it should be noted that the allocation of patients to systemic therapy groups was not randomized but rather influenced by physicians’ clinical judgment and patients’ disease severity. Such non-randomized treatment assignment may have introduced selection bias, particularly in subgroup comparisons, as patients with more advanced disease or poor prognostic features were more likely to receive systemic therapy. This factor should be considered when interpreting our findings, and prospective randomized studies are warranted to validate these observations. Beyond these clinical findings, emerging evidence indicates distinct molecular and immunological mechanisms that may underlie the synergy between IGTA and systemic therapy. Colorectal pulmonary metastases exhibit unique biological characteristics compared with hepatic or peritoneal lesions. Regressive mutations in KRAS and SMAD4, as well as specific ERBB2 point mutations, have been shown to influence tumor growth kinetics, metastatic behavior, and responsiveness to targeted or cytotoxic agents [24,25]. In addition, host immunogenetic factors—particularly certain HLA haplotypes—may enhance immune recognition of tumor-associated antigens, contributing to an oligometastatic phenotype with improved immune containment and treatment responsiveness [26]. These molecular and immunological determinants may partially explain why some patients derive greater benefit from the integration of IGTA and systemic therapy. Future translational research incorporating genomic and immunogenetic profiling could further elucidate these mechanisms and inform personalized multimodal treatment strategies.

Delayed IGTA were associated with shorter PFS and OS, reflecting a potential missed window for controlling CRLM. The no systemic therapy group demonstrated the poorest outcomes, underscoring the indispensable role of systemic agents in modern oncologic care. These findings align with the principles outlined by the European Society of Medical Oncology (ESMO), which advocate for systemic therapy as the cornerstone of metastatic CRC treatment [11,27].

Our study identified tumor number, size (≥3 cm), and peridiaphragmatic location as independent risk factors for LTP. Tumors near the diaphragm are prone to LTP due to respiratory motion during IGTA, complicating needle placement and ablation stability [28]. Thermal irritation may cause shoulder pain, limiting ablation power and completeness, while increasing risks of pneumothorax, pleural effusion, diaphragmatic injury, and adjacent organ damage [28,29].

Synchronous systemic therapy significantly mitigated the adverse prognostic impact of high-risk features such as synchronous and extrapulmonary metastases. This finding highlights the potential of early systemic therapy to address tumor heterogeneity and improve outcomes even in patients with aggressive disease biology. Future studies should explore whether combining IGTA with emerging systemic therapies, such as immune checkpoint inhibitors or next-generation anti-angiogenic agents, could further enhance outcomes. Preliminary evidence suggests that these agents not only modulate the tumor microenvironment but may also augment the abscopal effect of IGTA, expanding its therapeutic reach [10,11].

Our findings are consistent with the growing body of evidence supporting the integration of local and systemic therapies in metastatic CRC [30]. Wiesweg et al. demonstrated improved survival in patients treated with synchronized systemic therapy and local ablative therapy for pulmonary metastases, corroborating the benefits of coordinated multimodal approaches [31]. In studies on CRC liver metastases, upfront ablation combined with systemic therapy has shown significant improvements in DFS, highlighting the value of early intervention [12]. Although most evidence has focused on liver metastases, the parallels in survival benefits between pulmonary and hepatic metastases underscore the importance of optimizing treatment timing across metastatic sites.

Our study further demonstrates that mediastinal lymph node positivity (based on CT or pathology) is an independent risk factor for OS. Specifically, patients without lymph node metastasis have 3- and 5-year disease-specific survival rates of 73.5% and 58.3%, respectively, compared to 50.5% and 24.8% in those with confirmed lymph node metastasis [32]. This indicates a clear association between lymph node involvement and increased mortality risk, with mediastinal metastasis potentially conferring a worse prognosis than hilar lymph node involvement [33]. Our study also found that extrapulmonary metastases, particularly to bone, abdominal, or pelvic sites, significantly worsen OS, which is consistent with prior research. The prognosis for metastatic colorectal cancer is notably poorer when metastases occur in sites other than the liver and lungs, underscoring the need for comprehensive, systemic treatment strategies for these patients [34]. This observation also suggests a distinct biological behavior in such cases, which warrants further investigation to better understand the underlying mechanisms and to optimize therapeutic approaches. However, all participating centers in this study were located in China, which may limit the generalizability of our findings to other ethnic and healthcare settings. Differences in patient characteristics, treatment accessibility, and clinical practice patterns across regions could potentially influence outcomes. Therefore, external validation in broader, multinational, and ethnically diverse cohorts will be essential to confirm the robustness and applicability of our conclusions.

This study has several limitations that should be acknowledged. First, its retrospective cohort design may introduce selection bias, particularly in the allocation of patients to different systemic therapy groups. Although multivariate analyses adjusted for known confounders, residual bias cannot be entirely excluded. Second, a substantial subset of patients (*n* = 92) lacked systemic therapy records around the IGTA procedure. This reduced the robustness of subgroup comparisons and may have biased outcomes, underscoring the possibility of residual confounding despite statistical adjustments. Third, the heterogeneity of systemic therapy regimens across centers reflects real-world practice but complicates direct comparisons of treatment efficacy. Standardized protocols in future prospective studies would provide more robust evidence. Fourth, while our follow-up duration was adequate to capture most recurrences and survival events, longer-term outcomes remain uncertain, particularly in the context of emerging systemic therapies. Finally, the study focused on the timing and efficacy of systemic therapy and IGTA but did not delve into the specific impact of individual regimens or agents. While this was beyond the scope of the current study, future research should explore how different combinations of chemotherapy, targeted agents, and immunotherapies influence outcomes in CRLM.

## 5. Conclusion

This study highlights the critical importance of synchronizing systemic therapy with IGTA in the management of CRLM. Synchronous systemic therapy not only improves PFS and OS but also mitigates the adverse prognostic impact of high-risk features, such as synchronous and extrapulmonary metastases. These findings underscore the need for personalized treatment strategies that integrate systemic and local therapies in a time-sensitive manner. Future prospective studies, incorporating molecular profiling and advanced imaging, are essential to refine patient selection and optimize treatment sequencing.

## Supplementary Material

Sup.docx

## Data Availability

The datasets analyzed during the current study are available from the corresponding author upon reasonable request.
