# Supplementary material for: Management of potentially curable colorectal lung metastases with synchronous systemic therapy and percutaneous image-guided thermal ablation
Source: Ann Med. 2026 Jan 5;58(1):2612393. doi: 10.1080/07853890.2025.2612393 (PMC12777934; doi:10.1080/07853890.2025.2612393)
Supplement: Sup.docx [file IANN_A_2612393_SM4688.docx]

Table S1. Univariate and Multivariate Cox Regression Analysis of Local Tumor Progression-Free Survival (LTPFS)

| Variables | Univariate Analysis | | | Multivariate Analysis | |
| --- | --- | --- | --- | --- | --- |
|  | HR (95%CI) | | P | HR (95%CI) | P |
| Sex (male) | 1.211 (0.682, 2.149) | | 0.514 |  |  |
| Age (years) | 0.982 (0.955, 1.011) | | 0.227 |  |  |
| BMI (kg/m^2^) | 0.998 (0.922, 1.081) | | 0.968 |  |  |
| Comorbidities | 1.254 (0.711, 2.231) | | 0.434 |  |  |
| Extrapulmonary metastasis | 1.239 (0.653, 2.352) | | 0.512 |  |  |
| Primary tumor (ref. ascending colon) |  | | 0.007 |  |  |
| - Rectum | 0.373 (0.189, 0.734) | | 0.004 |  |  |
| - Sigmoid colon | 0.294 (0.124, 0.695) | | 0.005 |  |  |
| - Descending colon | 0.227 (0.075, 0.686) | | 0.009 |  |  |
| - Transverse colon | 0.252 (0.058, 1.103) | | 0.067 |  |  |
| Pneumoresection | 1.568 (0.703, 3.496) | | 0.271 |  |  |
| CEA > 20 ng/mL | 2.166 (1.105, 4.248) | | 0.024 |  |  |
| CA125 > 35 U/mL | 1.347 (0.418, 4.334) | | 0.618 |  |  |
| CA19-9 > 37 U/mL | 1.729 (0.837, 3.570) | | 0.139 |  |  |
| MWA (ref. RFA) | 0.922 (0.521, 1.631) | | 0.780 |  |  |
| Tumor number (ref. 1) |  | | <0.001 |  | <0.001 |
| - 2 tumors | 5.191 (1.722, 15.647) | | 0.003 | 3.918 (1.257, 12.212) | 0.019 |
| - 3 tumors | 15.531 (5.333, 45.224) | | <0.001 | 5.383 (1.647, 17.600) | 0.005 |
| - 4 tumors | 15.289 (4.454, 52.484) | | <0.001 | 13.882 (3.927, 49.071) | <0.001 |
| Laterality (ref. bilateral) |  | | <0.001 |  |  |
| - Left lung | 0.310 (0.152, 0.630) | | 0.001 |  |  |
| - Right lung | 0.219 (0.105, 0.457) | | <0.001 |  |  |
| Lung zone (ref. multiple) |  | | 0.009 |  |  |
| - Inner zone | 0.282 (0.084, 0.941) | | 0.039 |  |  |
| - Middle zone | 0.346 (0.148, 0.810) | | 0.014 |  |  |
| - Outer zone | 0.418 (0.220, 0.797) | | 0.008 |  |  |
| Tumor size > 3 cm | 6.143 (3.461, 10.902) | | <0.001 | 4.697 (2.404, 9.177) | <0.001 |
| Perivascular tumors | 1.262 (0.644, 2.474) | | 0.499 |  |  |
| Peribronchial tumors | 1.463 (0.729, 2.937) | | 0.285 |  |  |
| Near mediastinal pleura | 1.736 (0.885, 3.405) | | 0.109 |  |  |
| Near parietal pleura | 1.720 (0.972, 3.042) | | 0.061 |  |  |
| Peridiaphragmatic tumors | 3.900 (2.027, 7.506) | | <0.001 | 5.733 (2.873, 11.438) | <0.001 |
| Near interlobar fissure | 1.402 (0.782, 2.516) | | 0.257 |  |  |
| Systemic therapy (ref. none) | |  | 0.715 |  |  |
| - Delayed ablation | | 1.393 (0.632, 3.070) | 0.411 |  |  |
| - Synchronous ablation | | 1.162 (0.483, 2.795) | 0.737 |  |  |
| - Upfront ablation | | 1.585 (0.685, 3.671) | 0.282 |  |  |

**Note:** LTPFS, local tumor progression-free survival; BMI, body mass index; CEA, carcinoembryonic antigen; CA125, cancer antigen 125; CA19-9, carbohydrate antigen 19-9; MWA, microwave ablation; RFA, radiofrequency ablation.

Table S2 Univariate and Multivariate Cox Regression Analysis of Progression-Free Survival (PFS)

| Variables | Univariate Analysis | | Multivariate Analysis | |
| --- | --- | --- | --- | --- |
|  | HR (95%CI) | P | HR (95%CI) | P |
| Sex (male) | 1.000 (0.781, 1.280) | 0.998 |  |  |
| Age (years) | 1.011 (0.998, 1.024) | 0.085 |  |  |
| Pneumoresection | 0.978 (0.657, 1.457) | 0.978 |  |  |
| Primary tumor  (ref. ascending colon) |  | 0.018 |  |  |
| - Rectum | 0.649 (0.464, 0.908) | 0.012 |  |  |
| - Sigmoid colon | 0.514 (0.342, 0.771) | 0.001 |  |  |
| - Descending colon | 0.671 (0.435, 1.033) | 0.070 |  |  |
| - Transverse colon | 0.857 (0.503, 1.459) | 0.570 |  |  |
| Extrapulmonary metastasis  (ref. none) |  | <0.001 |  | 0.001 |
| - Liver | 1.541 (1.140, 2.081) | 0.005 | 1.480 (1.094, 2.002) | 0.011 |
| - Bone | 2.014 (1.138, 3.561) | 0.016 | 1.748 (0.976, 3.133) | 0.060 |
| - Abdominal cavity | 2.333 (1.457, 3.736) | <0.001 | 2.086 (1.256, 3.465) | 0.004 |
| - Pelvic cavity | 1.618 (0.964, 2.715) | 0.068 | 1.514 (0.882, 2.600) | 0.133 |
| - Other conditions | 2.396 (1.464, 3.922) | 0.001 | 2.576 (1.551, 4.278) | <0.001 |
| Systemic therapy (ref. none) |  | 0.006 |  | <0.001 |
| - Delayed ablation | 0.824 (0.609, 1.114) | 0.209 | 0.919 (0.673, 1.256) | 0.597 |
| - Synchronous ablation | 0.581 (0.410, 0.825) | 0.002 | 0.493 (0.341, 0.712) | <0.001 |
| - Upfront ablation | 0.610 (0.427, 0.872) | 0.007 | 0.563 (0.391, 0.810) | 0.002 |
|  |  |  |  |  |
| Synchronous metastases | 1.350 (1.033, 1.765) | 0.028 | 1.431 (1.067, 1.919) | 0.017 |
| CEA > 20 ng/mL | 1.078 (0.756, 1.538) | 0.678 |  |  |
| CA19-9 > 37 U/mL | 1.289 (0.918, 1.811) | 0.143 |  |  |
| MWA (ref. RFA) | 0.905 (0.711, 1.152) | 0.418 |  |  |
| T-stage (ref. T1) |  | 0.007 |  |  |
| - T2 | 3.800 (1.311, 11.015) | 0.014 |  |  |
| - T3 | 3.795 (1.391, 10.359) | 0.009 |  |  |
| - T4 | 4.988 (1.810, 13.749) | 0.002 |  |  |
| Tumor differentiation (ref. well) |  | 0.197 |  |  |
| - Poor | 1.370 (0.785, 2.391) | 0.267 |  |  |
| - Poor-to-moderate | 1.268 (0.816, 1.970) | 0.292 |  |  |
| - Moderate | 1.154 (0.784, 1.700) | 0.468 |  |  |
| - Moderate-to-well | 0.839 (0.538, 1.306) | 0.436 |  |  |
| Tumor size > 3 cm | 1.781 (1.341, 2.366) | <0.001 |  |  |
| Tumor number (ref. 1) |  | <0.001 |  | <0.001 |
| - 2 tumors | 1.723 (1.281, 2.317) | <0.001 | 1.675 (1.231, 2.280) | 0.001 |
| - 3 tumors | 2.132 (1.539, 2.952) | <0.001 | 1.861 (1.330, 2.603) | <0.001 |
| - 4 tumors | 3.404 (2.177, 5.323) | <0.001 | 3.821 (2.394, 6.099) | <0.001 |
| Laterality (ref. bilateral) |  | 0.134 |  |  |
| - Left lung | 0.755 (0.514, 1.108) | 0.151 |  |  |
| - Right lung | 0.681 (0.467, 0.992) | 0.046 |  |  |
| Lung field (ref. multiple) |  | 0.550 |  |  |
| - Central | 0.895 (0.388, 2.065) | 0.795 |  |  |
| - Peripheral | 0.752 (0.354, 1.597) | 0.459 |  |  |
| Emphysema | 1.099 (0.791, 1.528) | 0.574 |  |  |
| Perivascular tumors | 0.865 (0.640, 1.168) | 0.343 |  |  |
| Peribronchial tumors | 1.008 (0.727, 1.397) | 0.963 |  |  |
| Near mediastinal pleura | 1.348 (0.986, 1.843) | 0.061 |  |  |
| Near parietal pleura | 1.386 (1.089, 1.763) | 0.008 |  |  |
| Hilar lymph nodes (+) | 0.819 (0.572, 1.174) | 0.278 |  |  |
| Mediastinal lymph nodes (+) | 1.922 (1.415, 2.612) | <0.001 |  |  |
| DFI (<24 months) | 1.323 (1.041, 1.682) | 0.022 |  |  |
| Interval between primary res-ction and IGTA (<24 months) | 1.234 (0.967, 1.573) | 0.091 |  |  |
| Peridiaphragmatic tumors | 1.195 (0.792, 1.805) | 0.396 |  |  |
| Near interlobar fissure | 1.235 (0.958, 1.593) | 0.103 |  |  |

**Note:** PFS, progression-free survival; CEA, carcinoembryonic antigen; CA19-9, carbohydrate antigen 19-9; MWA, microwave ablation; RFA, radiofrequency ablation; DFI, disease-free interval from primary resection to the diagnosis of liver metastasis; IGTA, image-guided thermal ablation.

Table S3 Univariate and multivariate Cox regression analysis for Overall Survival (OS)

| Variables | Univariate Analysis | | Multivariate Analysis | |
| --- | --- | --- | --- | --- |
|  | HR (95%CI) | P | HR (95%CI) | P |
| Sex (male) | 1.010 (0.742, 1.375) | 0.950 |  |  |
| Age (years) | 1.009 (0.994, 1.024) | 0.253 |  |  |
| Pneumoresection | 1.155 (0.708, 1.883) | 0.565 |  |  |
| Primary tumor  (ref. ascending colon) |  | <0.001 |  |  |
| - Rectum | 0.690 (0.469, 1.014) | 0.059 |  |  |
| - Sigmoid colon | 0.379 (0.230, 0.624) | <0.001 |  |  |
| - Descending colon | 0.328 (0.183, 0.590) | <0.001 |  |  |
| - Transverse colon | 0.534 (0.266, 1.073) | 0.078 |  |  |
| Extrapulmonary metastasis  (ref. none) |  | <0.001 |  | <0.001 |
| - Liver | 1.226 (0.808, 1.861) | 0.338 | 1.255 (0.824, 1.910) | 0.290 |
| - Bone | 4.500 (2.438, 8.304) | <0.001 | 3.906 (2.076, 7.350) | <0.001 |
| - Abdominal cavity | 4.742 (2.858, 7.868) | <0.001 | 5.837 (3.423, 9.953) | <0.001 |
| - Pelvic cavity | 2.805 (1.610, 4.888) | <0.001 | 3.231 (1.834, 5.692) | <0.001 |
| - Other conditions | 6.764 (4.022, 11.376) | <0.001 | 7.516 (4.217, 13.397) | <0.001 |
|  |  |  |  |  |
| Synchronous metastases | 1.679 (1.219, 2.311) | 0.001 |  |  |
| CEA > 20 ng/mL | 1.026 (0.655, 1.605) | 0.912 |  |  |
| CA19-9 > 37 U/mL | 1.669 (1.124, 2.478) | 0.011 |  |  |
| MWA (ref. RFA) | 0.780 (0.578, 1.052) | 0.104 |  |  |
| T-stage (ref. T1) |  | 0.462 |  |  |
| - T1 | 1.521 (0.498, 4.644) | 0.462 |  |  |
| - T2 | 1.986 (0.727, 5.429) | 0.181 |  |  |
| - T3 | 1.965 (0.709, 5.451) | 0.194 |  |  |
| Tumor differentiation (ref. well) |  | 0.276 |  |  |
| - Poor | 1.689 (0.850, 3.354) | 0.135 |  |  |
| - Poor-to-moderate | 1.614 (0.916, 2.844) | 0.097 |  |  |
| - Moderate | 1.139 (0.678, 1.914) | 0.623 |  |  |
| - Moderate-to-well | 1.303 (0.735, 2.308) | 0.365 |  |  |
| Tumor size > 3 cm | 2.596 (1.874, 3.596) | <0.001 | 1.512 (1.032, 2.216) | 0.034 |
| Tumor number (ref. 1) |  | <0.001 |  | 0.002 |
| - 2 tumors | 1.694 (1.138, 2.522) | 0.009 | 1.457 (0.969, 2.191) | 0.071 |
| - 3 tumors | 3.548 (2.385, 5.280) | <0.001 | 2.068 (1.306, 3.274) | 0.002 |
| - 4 tumors | 3.086 (1.802, 5.285) | <0.001 | 2.700 (1.526, 4.778) | 0.001 |
| Laterality (ref. bilateral) |  | <0.001 |  |  |
| - Left lung | 0.391 (0.252, 0.607) | <0.001 |  |  |
| - Right lung | 0.446 (0.292, 0.679) | <0.001 |  |  |
| Lung field (ref. multiple) |  | 0.173 |  |  |
| - Central | 0.542 (0.220, 1.331) | 0.181 |  |  |
| - Peripheral | 0.487 (0.228, 1.042) | 0.064 |  |  |
| Emphysema | 1.096 (0.726, 1.654) | 0.662 |  |  |
| Perivascular tumors | 1.144 (0.791, 1.653) | 0.475 |  |  |
| Peribronchial tumors | 1.284 (0.874, 1.885) | 0.203 |  |  |
| Near mediastinal pleura | 1.557 (1.073, 2.258) | 0.020 |  |  |
| Near parietal pleura | 1.093 (0.809, 1.476) | 0.564 |  |  |
| Hilar lymph nodes (+) | 1.119 (0.741, 1.688) | 0.593 |  |  |
| Mediastinal lymph nodes (+) | 2.365 (1.666, 3.357) | <0.001 | 1.518 (1.034, 2.228) | 0.033 |
| DFI (<24 months) | 1.756 (1.299, 2.375) | <0.001 |  |  |
| Interval between primary res-ction and IGTA (<24 months) | 1.636 (1.211, 2.209) | 0.001 |  |  |
| ITGA timing (vs. none) |  | <0.001 |  | <0.001 |
| - Delayed ablation | 0.662 (0.456, 0.961) | 0.030 | 0.589 (0.402, 0.863) | 0.007 |
| - Synchronous ablation | 0.384 (0.240, 0.612) | <0.001 | 0.211 (0.127, 0.352) | <0.001 |
| - Upfront ablation | 0.543 (0.350, 0.841) | 0.006 | 0.466 (0.297, 0.731) | 0.001 |
| Peridiaphragmatic tumors | 1.247 (0.765, 2.032) | 0.376 |  |  |
| Near interlobar fissure | 1.331 (0.974, 1.820) | 0.073 |  |  |

**Note:** OS, overall survival; CEA, carcinoembryonic antigen; CA19-9, carbohydrate antigen 19-9; MWA, microwave ablation; RFA, radiofrequency ablation; DFI, disease-free interval from primary resection to the diagnosis of liver metastasis; IGTA, image-guided thermal ablation.
